# OTX2 Non-Cell Autonomous Activity Regulates Inner Retinal Function

**DOI:** 10.1523/ENEURO.0012-19.2020

**Published:** 2020-09-02

**Authors:** Raoul Torero Ibad, Bilal Mazhar, Clémentine Vincent, Clémence Bernard, Julie Dégardin, Manuel Simonutti, Thomas Lamonerie, Ariel A. Di Nardo, Alain Prochiantz, Kenneth L. Moya

**Affiliations:** 1 Centre for Interdisciplinary Research in Biology (CIRB), Collège de France, CNRS, UMR 7241, INSERM U1050, Labex MemoLife, Université PSL (Paris Sciences & Lettres), 75005 Paris, France; 2 CNRS, INSERM, Institut de la Vision, Sorbonne Université, F-75012 Paris, France; 3 CNRS, INSERM, Institut de Biologie Valrose, Université Côte d’Azur, 06088 Nice, France; 4 BrainEver, 75012 Paris, France; 5 Institute of Neurosciences, Chinese Academy of Sciences, Shanghai 200031, People’s Republic of China

**Keywords:** homeoprotein non-cell autonomous, physiology retina, vision

## Abstract

OTX2 is a homeoprotein transcription factor expressed in photoreceptors and bipolar cells in the retina. OTX2, like many other homeoproteins, transfers between cells and exerts non-cell autonomous effects such as promoting the survival of retinal ganglion cells that do not express the protein. Here we used a genetic approach to target extracellular OTX2 in the retina by conditional expression of a secreted single-chain anti-OTX2 antibody. Compared with control mice, the expression of this antibody by parvalbumin-expressing neurons in the retina is followed by a reduction in visual acuity in 1-month-old mice with no alteration of the retinal structure or cell type number or aspect. The a-waves and b-waves measured by electroretinogram were also indistinguishable from those of control mice, suggesting no functional deficit of photoreceptors and bipolar cells. Mice expressing the OTX2-neutralizing antibody did show a significant doubling in the flicker amplitude and a reduction in oscillatory potential, consistent with a change in inner retinal function. Our results show that interfering *in vivo* with OTX2 non-cell autonomous activity in the postnatal retina leads to an alteration in inner retinal cell functions and causes a deficit in visual acuity.

## Significance Statement

OTX2 is a homeoprotein transcription factor expressed in retinal photoreceptors and bipolar cells. Although the *Otx2* locus is silent in the inner retina, the protein is detected in cells of the ganglion cell layer consistent with the ability of this class of proteins to transfer between cells. We expressed a secreted single chain antibody (scFv) against OTX2 in the retina to neutralize extracellular OTX2. Antibody expression leads to reduced visual acuity with no change in retinal structure, or photoreceptor or bipolar physiology; however, activity in the inner retina was altered. Thus, interfering with OTX2 non-cell autonomous activity in postnatal retina alters inner retinal function and causes vision loss, highlighting the physiological value of homeoprotein direct non-cell autonomous signaling.

## Introduction

OTX2 is a homeoprotein transcription factor important for retinal development and maintenance. It is expressed early in the embryonic mouse optic vesicle and retinal pigmented epithelium (RPE) and is required for differentiation of photoreceptors by transactivation of *Crx* and *Nrl* and for differentiation of bipolar cells via regulation of PKCα (Martinez-Morales et al., 2001; Nishida et al., 2003; Koike, et al., 2007; Muranishi et al., 2011). In mice hypomorph for *Otx2*, visual deficits, retinal physiological dysfunction and age- and OTX2-dependent degeneration of the retina have been reported (Bernard et al., 2014). In adult mice, knockdown of *Otx2* in RPE results in photoreceptor death, demonstrating that continued expression of OTX2 in RPE is necessary for photoreceptor survival (Housset et al., 2013). Exogenous OTX2 protects adult retinal ganglion cells (RGCs) against NMDA-induced excitotoxicity and preserves visual acuity (Torero Ibad et al., 2011).

The capacity of homeoproteins (HPs) to transfer between cells allows different types of activities. Homeoproteins can act within the cells that produce them, thus in a cell autonomous fashion, but they can also exert their activity extracellularly or by transferring to cells that do or do not produce them, i.e., non-cell autonomously. Two separate sequences necessary and sufficient for HP cell exit and entry are in the DNA-binding homeodomain (for review, see Di Nardo et al., 2018). A simple genetic approach cannot therefore be used to study their direct non-cell autonomous activity, as mutation of either sequence alters OTX2 DNA binding and thus also alters cell autonomous activities. An alternative genetic approach was used to specifically target extracellular OTX2 to only abolish non-cell autonomous activity. Conditional mice have been designed to express a neutralizing secreted anti-OTX2 single chain antibody (*scFvOTX2^tg/o^* mice) in a Cre-dependent manner (Bernard et al., 2016). In the retina parvalbumin (PV) is only expressed by RGCs and amacrine cells that do not express *Otx2*. Thus it is anticipated that in *PV^Cre^*::*scFvOTX2^tg/o^* mice, OTX2-scFv expressed and secreted from RGCs and amacrine cells will sequester extracellular OTX2 in the vicinity of the producing cells, thus blocking its non-cell autonomous activities. This strategy based on anti-HP scFv *in vivo* secretion has been used with success in several animal models to neutralize extracellular PAX6, ENGRAILED, and OTX2 (Lesaffre et al., 2007; Wizenmann et al., 2009; Layalle et al., 2011; Bernard et al., 2016).

We show here that the sequestration of extracellular OTX2 by the OTX2-scFv secreted by RGCs and amacrine cells leads to a significant decrease in visual acuity. This decrease takes place in absence of any observable developmental defects, laminar abnormalities or changes in cell lineages. Electroretinogram (ERG) measurements show normal outer and inner nuclear function but show a twofold increase in amplitude in the response to 20 Hz flickers. Together, our results provide evidence for a direct non-cell autonomous activity of OTX2 for RGC function.

## Materials and Methods

*Production of transgenic mice. scFvOTX2^tg/o^* and *scFvPAX6^tg/o^* mice were produced by the Institut Clinique de la Souris (Strasbourg, France) as described previously (Bernard et al., 2016). The mice were crossed with *PV^Cre^* mice obtained from The Jackson Laboratory (stock #8069). Mice were used without regard to sex, and males and females were used in all experiments. All animal experiments were conducted in accordance with European Directive number 86/609 (EEC Council for Animal Protection in Experimental Research and Other Scientific Utilization) and French authorization n°00702.01, “Viellissement, dégénération et régénération du système nerveux central adulte chez la souris,” delivered by the French Ministère de l’Enseignement Supérieur et de la Recherche.

*Immunoprecipitation and Western blot.* Immunoprecipitation and Western blotting were conducted as described previously (Bernard et al., 2016). Retinas were dissected and suspended in immunoprecipitation lysis buffer (20 mm Tris pH 8, 120 mm NaCl, 1% NP-40, 1 mm MgCl_2_, 5% glycerol, Benzonase Nuclease, and protease inhibitors). Samples were centrifuged (10 min, 20,000 × *g*) at 4°C, and the supernatants incubated overnight at 4°C, with rotation with anti-GFP-coupled magnetic beads (Chromotek). The beads were washed with lysis buffer and 1 m urea before Western blot analysis. Protein extracts were separated on an Invitrogen NuPAGE 4–12% Bis-Tris precast gel (Thermo Fisher Scientific) for 1 h at 200 V and transferred onto a methanol-activated PVDF membrane at 400 mA for 1 h. The membrane was blotted with an anti-MYC antibody (rabbit polyclonal, 1/4000; catalog #C3956, Sigma-Aldrich) and imaged with a LAS-4000 gel imager (Fujifilm).

In situ *hybridization for OTX2scFV expression. In situ* hybridization (ISH) was performed on retinal sections using RNAscope technology. Eyes were removed and fixed overnight at 4°C in buffered 4% paraformaldehyde (PFA) and cryoprotected in 20% sucrose overnight at 4°C. Forty micrometer cryostat sections were collected on Superfrost slides, dried, and stored at −20°C. On the day of processing, they were thawed to room temperature for 15 min before performing ISH according to the protocol of the manufacturer (Advanced Cell Diagnostics). Briefly, endogenous peroxidase was neutralized with H_2_O_2_, permeabilized with buffered Tween 20 using reagents provided by the manufacturer. Probes designed by Advanced Cell Diagnostics were hybridized for 2 h at 40°C, and the signal amplified in three steps was visualized either with Red (570 nm) or Far Red (690 nm). The probes *PV* (Mm-PV-C2) and *Myc* (Mm-MYC-C1) were used at 1:50 dilution.

*Visual acuity.* The optomotor test of visual acuity was used to screen for possible phenotype differences. Postnatal day 30 (P30) mice were used since it was thought that this age would allow sufficient time for scFv expression and secretion. The optomotor test was performed as described in the literature with an optotype of 0.375c/deg, which is known to elicit robust responses (Torero Ibad et al., 2011; Bernard et al., 2014). Briefly, mice were placed on an elevated platform centered in a rotating drum, and a square-wave 100% contrast optotype of 0.375c/deg was rotated at a speed of 2 rpm. Mice were filmed from above and scored in real time by two observers blind to genotype for head turns in the direction and speed of the moving grating. Any discrepancies between the two observers’ real-time score was resolved by analyzing the video. A Mann–Whitney *U* test was used to compare the genotypes because of unequal variances.

*Retinal histology.* Eyes were removed and placed in 4% PFA, 2% ZnCl, and 20% isopropyl alcohol (Bernard et al., 2014). Five micrometer paraffin sections were cut in the vicinity of the optic nerve and stained for hematoxylin and eosin (Excalibur Pathology, Inc.). Three sections per retina were analyzed per animal. Total number of cells in the entire ganglion cell layer (GCL; i.e., RGCs and displaced amacrine cells) were counted for each of three sections that included the optic nerve head and averaged. The thickness of the inner nuclear layer (INL) and outer nuclear layer (ONL) proximal to the optic nerve head was measured on the three sections per animal and averaged.

*Quantitative PCR for OTX2scFv expression and retinal cell types.* RNA was extracted from frozen retinas of P30 mice with RNeasy Lipid Tissue Mini Kit (Qiagen). cDNA was generated with the Quantitect Reverse transcription kit (Qiagen). Using the 2^-ΔΔCt^ method, sample expression levels were normalized to *hprt* and to *PV^Cre^* mice levels. Primers for the following cell types were used: *Otx2-scFv* (transgene expression), *Brn3A* (ganglion cells), *Chx10* (bipolar cells), *Syntaxin* (amacrine and horizontal cells), *Lim1* (horizontal cells), *Rhodopsin* (rods), and *Red Opsin* (L/M cone cells). Since the data are normalized to the value for PVCre mice, the nonparametric Mann–Whitney *U* test was used to evaluate the difference for *Lim1* expression.

*Electroretinogram.* Mice were anesthetized with xylazine/ketamine (100 mg/kg, Imalgene 500, Virbac; 10 mg/kg, Rompun 2%, Bayer). Tropicamide (Mydriaticum 0.5%, Théa) was used to dilate the pupil, and the cornea was locally anesthetized with oxybuprocaine hydrochloride. Gold electrodes were placed in contact with each eye, reference electrodes were placed subcutaneously in the submandibular area, and a ground electrode was placed subcutaneously on the back of the animal. ERG was performed with a mobile apparatus (SIEM Bio-Medical) with LED lamps in a Ganzfeld chamber controlled by the VisioSystem software. ERG recordings were obtained from seven *PV^Cre^* and nine *PV-Cre::OTX2-scFv* mice 30–31 d of age. The right and left eye of each mouse was considered to be independent. A Mann–Whitney *U* test was used to evaluate the difference between genotypes for 20 Hz flickers and oscillatory potential.

## Results

**Figure 1.**
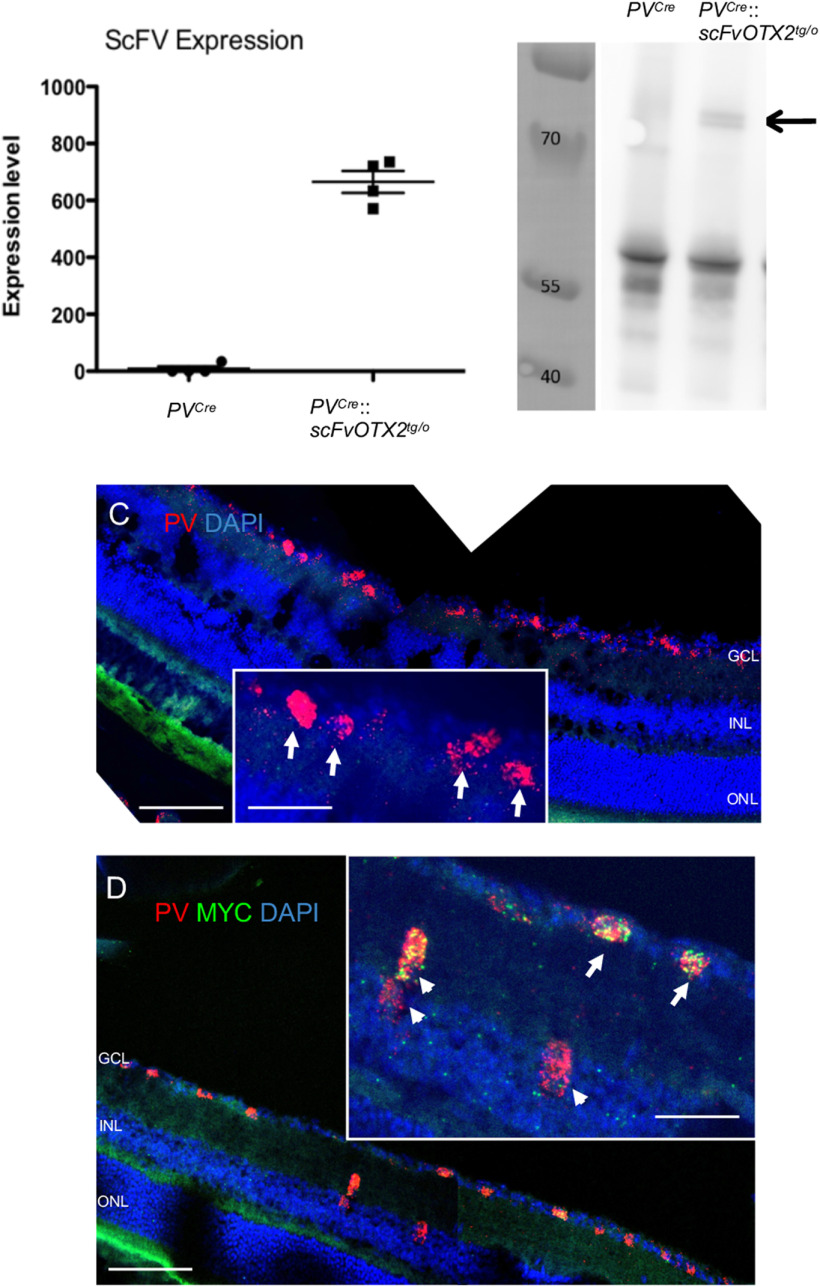
Expression of OTX2-scFV in P30 mouse retinal tissue. ***A***, qRT-PCR for *OTX2scFV* mRNA was conducted on extracts from *PV^Cre^* and *PV^Cre^*::*scFvOTX2^tg/o^* mice and raw ct values normalized and converted to arbitrary units. ***B***, Retinal lysates from *PV^Cre^* and *PV^Cre^*::*scFvOTX2^tg/o^* mice were immunoprecipitated for GFP followed by Western blotting with an anti-MYC antibody. Molecular weight markers are in the left lane in kDa. The middle lane contains the lysate from *PV^Cre^* mice. Right lane contains lysate from *PV^Cre^*::*scFvOTX2^tg/o^* mice. The arrow indicates the presence of the bands at the expected migration position of the full-length OTX2scFv-GFP protein. ***C***, *In situ* hybridization using RNAscope technology of *PV^Cre^* retina shows PV expression (red) in the inner retina. DAPI is in blue. Inset arrows show details of PV-expressing cells in the GCL. ***D***, *In situ* hybridization using RNAscope technology of *PV^Cre^*::*scFvOTX2^tg/o^* retina. mRNA for *PV* is red, and mRNA for the myc tag of the *OTX2scfv* is green. Arrows indicate scFv-expressing PV cells in the GCL (i.e., displaced amacrines and/or RGCs); arrowheads indicate scFv-expressing PV cells in the innermost part of the INL in the place of amacrine cells. Scale bars: low-magnification images, 100 μm; insets, 50 μm. Values: mean ± sem.

In the retina, PV is expressed in RGCs and amacrine cells (Haverkamp and Wässle, 2000; Haverkamp et al., 2009). As shown in Figure 1*A*, quantitative real-time PCR (qRT-PCR) shows significant expression of the *OTX2-scFv* transgene in the retinas of *PV^Cre^*::*scFvOTX2^tg/o^* mice and no detectable expression in the retinas of *PV^Cre^* mice (raw ct *PVCre *=* *38.2 ± 1.7; *PV::Cre*;*scFvOTX2^tg/o^* = 28.9 ± 0.1). Because the antibody is fused with GFP (Bernard et al., 2016), we immunoprecipitated the OTX2-scFv protein from *PV^Cre^*::*scFvOTX2^tg/o^* retina using an anti-GFP antibody followed by Western blot analysis. We detected a doublet at the expected 78 kDa molecular weight, that was not present in extracts from retina of *PV^Cre^* control mice (Fig. 1*B*, arrow). *In situ* hybridization (Fig. 1*C*) revealed PV-expressing cells in the inner retina in the reported location of PV-containing cells (Haverkamp and Wässle, 2000). In *PV^Cre^*::*scFvOTX2^tg/o^* retinas, each PV-expressing cell also expressed mRNA for the scFv in *PV*-expressing cells, as expected (Fig. 1*D*). This demonstrates the expression of the transgene in PV cells and the presence of full-length OTX2-scFv protein in the retinas of *PV^Cre^*::*scFvOTX2^tg/o^* mice.

**Figure 2.**
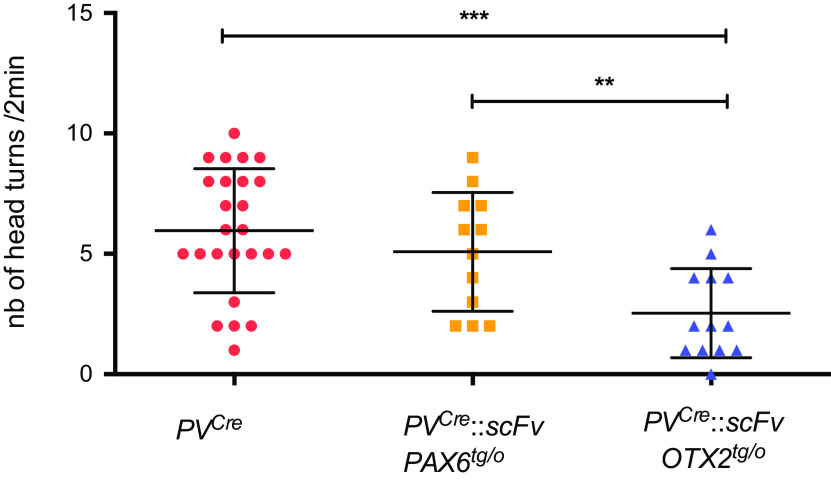
Optomotor test monitoring visual acuity. Thirty-day-old mice were subjected to the optomotor test using an 100% contrast optotype of 0.375c/deg. *PV^Cre^* and *PV^Cre^*::*scFvPax6^tg/o^* mice made an average of about five head turns during the 2 min test period. *PV^Cre^*::*scFvOTX2^tg/o^* mice made significantly fewer head turns revealing reduced visual acuity. **p < 0.01 or ***p < 0.001, Mann–Whitney *U* test, two tailed. *N* = 25 for *PV^Cre^*, *N* = 12 for *PV^Cre^*::*scFvPax6^tg/o^*, and *N* = 13 for *PV^Cre^*::*scFvOTX2^tg/o^*. Values: mean ± sem.

One-month-old mice were tested for visual acuity by examining their optokinetic response to a 100% contrast optotype square wave grating of 0.375c/deg spatial frequency. Bernard et al. (2014) reported that this spatial frequency was most robust in revealing differences in mice hypomorph for OTX2 activity, and the number of head movements in response to this optotype is sensitive to changes in visual acuity because of loss of RGCs (Torero Ibad et al., 2011). A significant reduction in visual performance in the *PV^Cre^*::*scFvOTX2^tg/o^* mice was observed compared with *PV^Cre^* littermates (Fig. 2, Table 1). As an additional control, we also evaluated *PV^Cre^*::*scFvPAX6^tg/o^* mice. PAX6 homeoprotein is reported to also have non-cell autonomous activities (Di Lullo et al., 2011; Kaddour et al., 2019), and these mice showed no visual acuity loss. These results demonstrate that neutralizing extracellular OTX2 in the retina leads to decreased visual acuity at P30, but the present results do not allow us to determine whether this persists at later ages.

**Table 1:** Statistical table

Data structure	Type of test	Power
Figure 2 *PV^cre^*vs *PVCre*::*scFvOTX2*^tg/o^ Not considered normal due to unequal *N*	Mann–Whitney *U*	0.92
Figure 4 *PV^cre^*vs *PVCre*::*scFvOTX2*^tg/o^ Data normalized	Mann–Whitney *U*	0.78
Figure 5 *PV^cre^*vs *PVCre*::*scFvOTX2*^tg/o^ a-wave latency, *F*_(1,14)_ = 0.011 a-wave amplitude, *F*_(1,14)_ = 0.100 b-wave latency, *F*_(1,14)_ = 3.826 b-wave amplitude, *F*_(1,14)_ = 0.77 Genotype × stimulus intensity interaction a-wave latency, *F*_(3,42)_ = 0.660 a-wave amplitude, *F*_(3,42)_ = 0.913 b-wave latency, *F*_(3,41)_ = 1.915 b-wave amplitude, *F*_(3,42)_ = 0.139,	Two-way ANOVA with repeated measures	n.s. n.s. n.s. n.s. n.s. n.s. n.s. n.s.
Figure 7 OPs *PV^cre^*vs *PVCre*::*scFvOTX2*^tg/o^ Dissimilar variance Figure 7*D* Flickers *PV^cre^*vs *PVCre*::*scFvOTX2*^tg/o^ Dissimilar variance	Mann–Whitney *U* Mann–Whitney *U*	0.97 0.96

**Figure 3.**
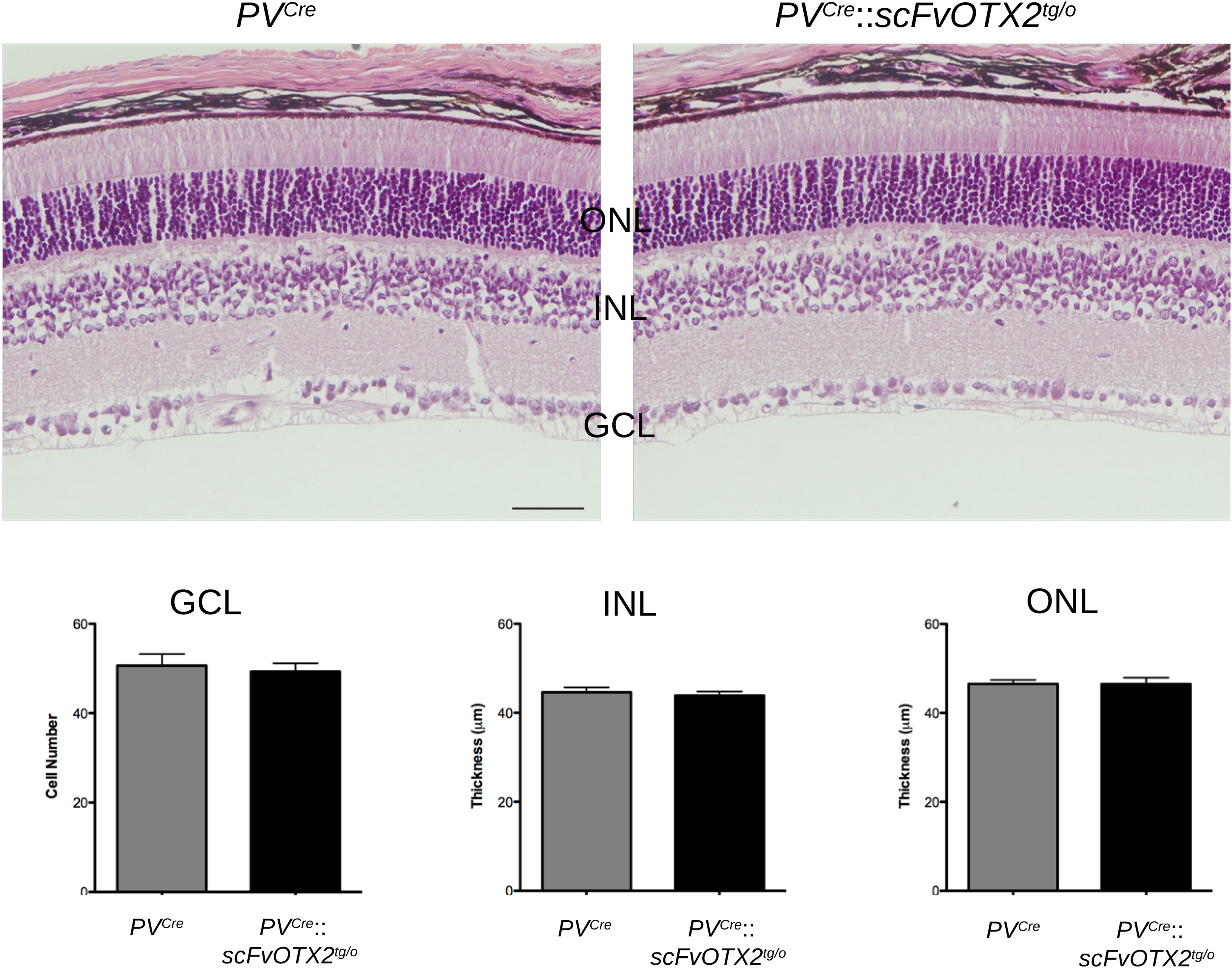
Expression of OTX2scFv does not alter retinal organization in the P30 mouse. Top, All retinal layers organization and approximate sizes are similar in retina from mice expressing OTX2scFv and their *PV^Cre^* littermates. Bottom, The number of cells in the GCL, the INL thickness, and the ONL thickness are similar between the two genotypes. Scale bar, 50 μm. *N* = 8 for *PV^Cre^* mice and *N* = 6 for *PV^Cre^*::*scFvOTX2^tg/o^* mice. Values: mean ± sem.

In search of a cellular correlate, we analyzed retinal structure by hematoxylin and eosin staining of sections from 1-month-old mice. There was no difference in thickness of the ONL (photoreceptors) or of the INL (horizontal, bipolar, amacrines, and Müller glial cells) between *PV^Cre^*::*scFvOTX2^tg/o^* and *PV^Cre^* littermates (Fig. 3). Nor could we find a difference in the number of cells in the GCL containing RGCs and displaced amacrine cells. These results thus show no gross structural abnormalities in the retinas of *PV^Cre^*::*scFvOTX2^tg/o^* mice or obvious differences in the number of cells in the ONL, INL, or GCL.

**Figure 4.**
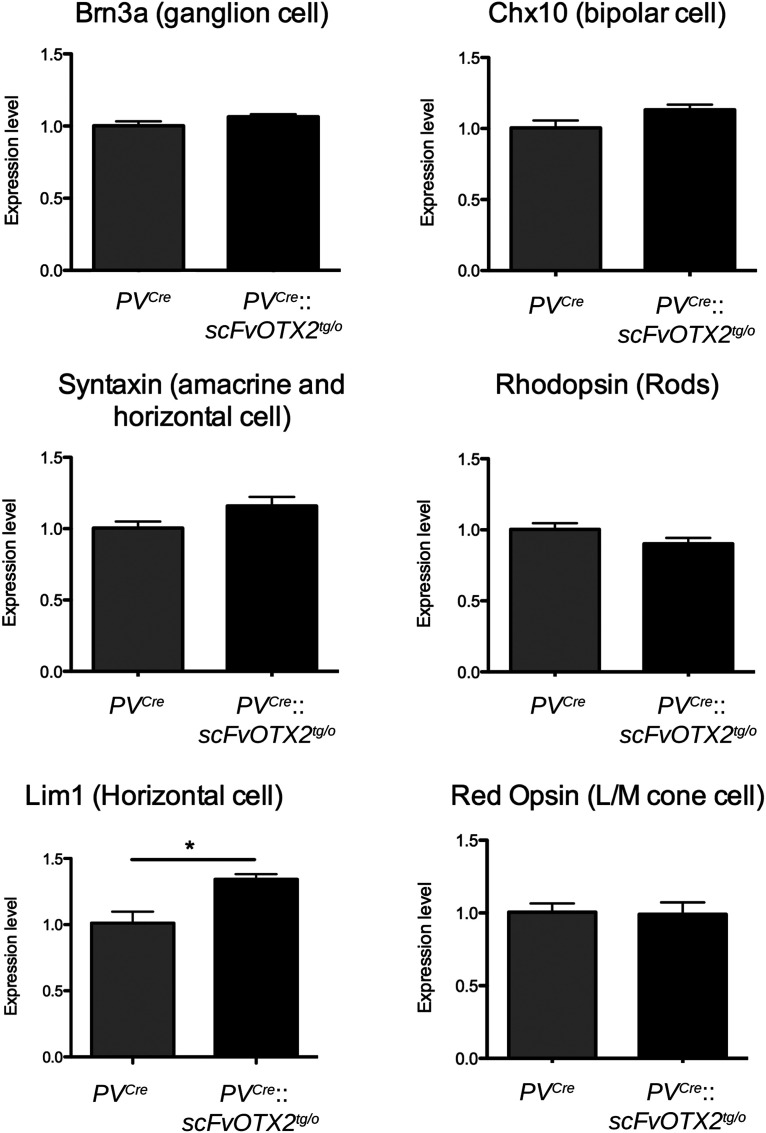
Expression of retinal cell type-specific genes in P30 mice of the two genotypes. Genes specific of RGCs, bipolar cells, amacrine cells, rods, and cones show similar expression levels in the two genotypes. *Lim1* mRNA expression in horizontal cells is significantly increased in the OTX2-scFv-expressing mice. *p < 0.05, Mann–Whitney *U* test, two tailed. *N* = 4 for each genotype. Values: mean ± sem.

Quantitative RT-PCR was reported to faithfully reflect retinal cell number (Torero Ibad et al., 2011). We observed no differences between P30 *PV^Cre^*::*scFvOTX2^tg/o^* and *PV^Cre^* littermates for the expression of mRNAs for *Brn3A* (RGCs), *syntaxin* (amacrine and horizontal cells), *Chx10* (bipolar cells), *rhodopsin* (rod photoreceptors), and *red/green opsin* (long/medium-wavelength cone photoreceptors; Fig. 4). A small increase in *Lim1* expression by horizontal cells was observed that might reflect a non-cell autonomous repressor activity of OTX2 on *Lim1* expression (Nakano et al., 2000; Puelles et al., 2006). Thus, based on these markers, histologic studies, and cell counting, it can be concluded that all cell types involved in retinal visual processing are present in normal numbers.

**Figure 5.**
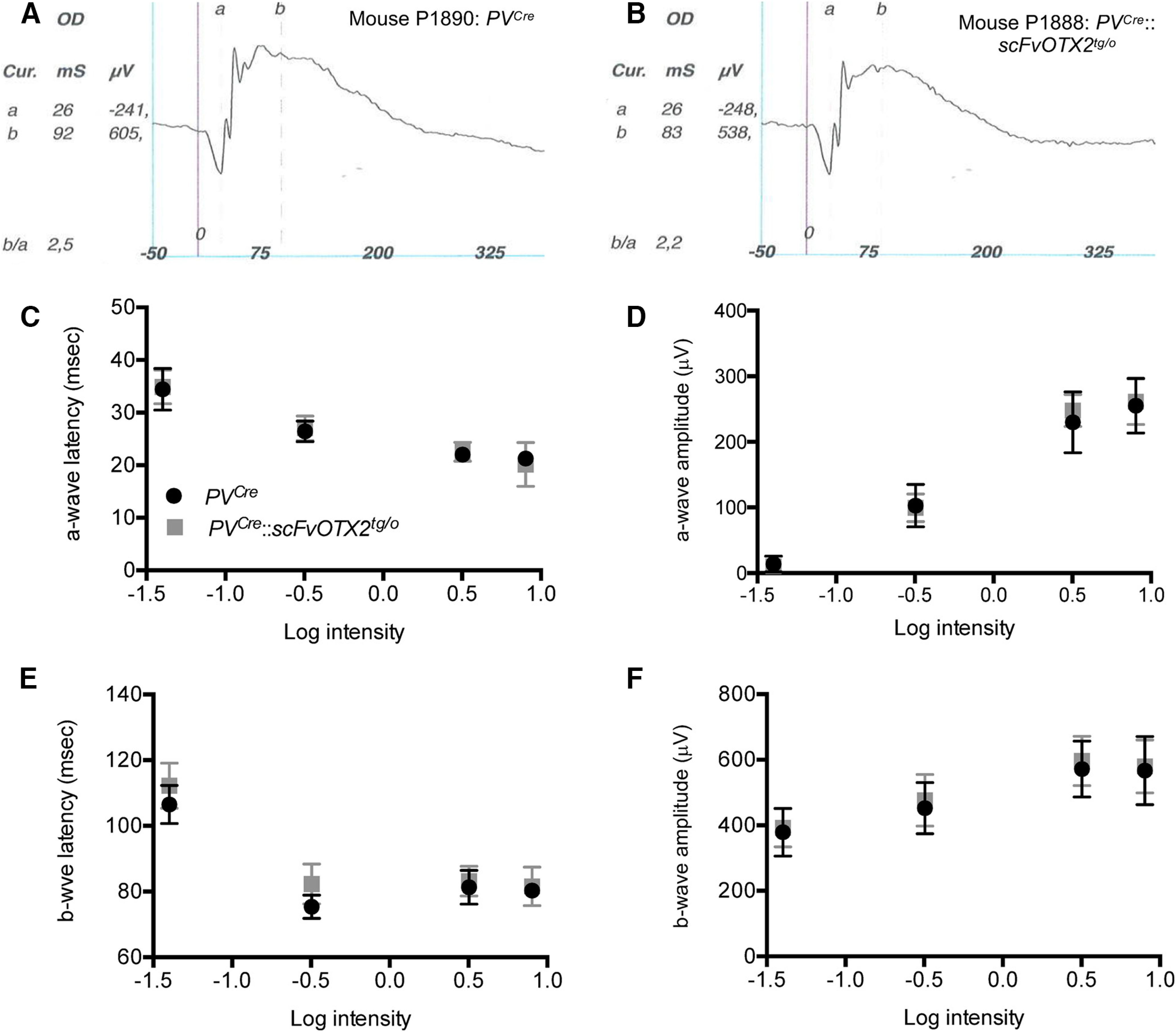
ERG of P30 mice expressing OTX2scFV and their PV^Cre^ littermates. ***A***, ***B***, Representative traces under scotopic conditions of the right eye at 3.19 cd/m^2^. ***C–F***, Scotopic ERG parameters plotted against log intensity. Two-way ANOVA for repeated measures showed no significant differences based on genotype or genotype by intensity interaction (see text). *N* = 7 for *PV^Cre^* mice and *N* = 9 for *PV^Cre^::scFvOTX2^tg/o^* mice. Values: mean ± sd.

**Figure 6.**
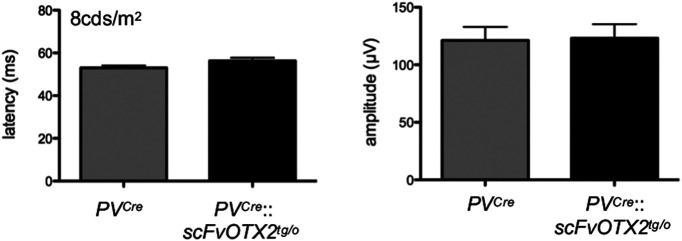
The b-wave amplitude in photopic lighting in the two genotypes of 1-month-old mice. *N* = 7 for *PV^Cre^* mice and *N* = 9 for *PV^Cre^*::*scFvOTX2^tg/o^* mice. Values: mean ± sem.

**Figure 7.**
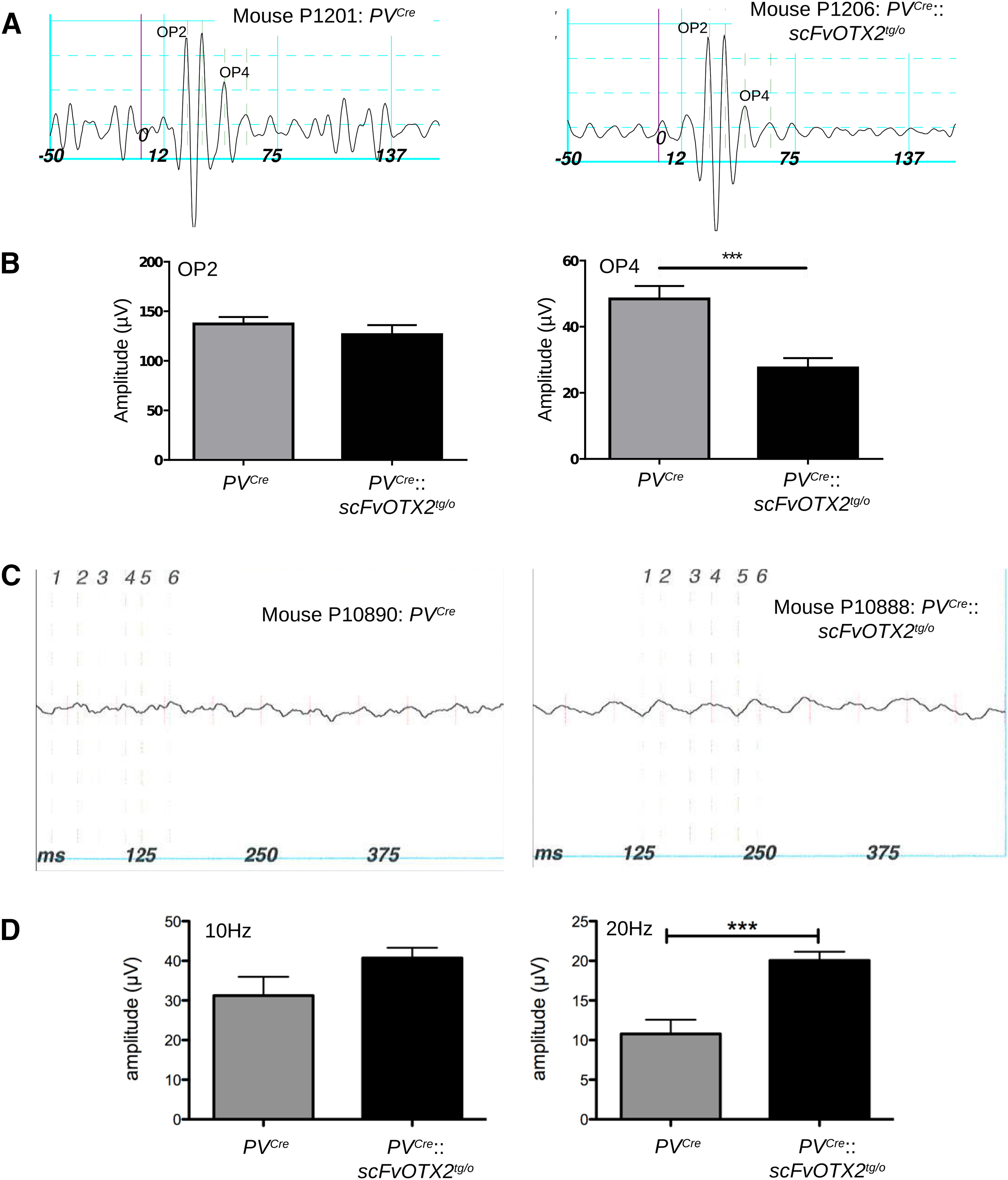
Inner retinal function. ***A***, Extracted oscillatory potential traces of a *PV^Cre^* mouse (left) and *PV^Cre^*::*scFvOTX2^tg/o^* mouse (right). ***B***, The amplitude of an early OP (OP2) is not altered in the *PV^Cre^*::*scFvOTX2^tg/o^* mice, while the amplitude of a late OP (OP4) is significantly reduced by ∼50%. ***C***, Representative traces of 20 Hz flickers of the left eye of mice of the two genotypes. ***D***, The amplitude of the flicker response to 10 Hz stimulation after light adaptation is slightly increased, and at 20 Hz the response is significantly doubled in amplitude. Bottom, There was no difference in the implicit time of the flicker responses between mice expressing OTX2scFv and their *PV^Cre^* littermates. ***p < 0.005, Mann–Whitney *U* test, two tailed. *N* = 7 for *PV^Cre^* and *N* = 9 for *PV^Cre^*::*scFvOTX2^tg/o^* mice. Values mean ± sem.

To determine whether there was a functional defect in photoreceptors and/or bipolar cells, we turned to ERG. The negative a-wave reflects photoreceptor hyperpolarization in response to light. The amplitude is related to photoreceptor number and the implicit time to photoreceptor physiology (Hood and Birch, 1992; Lamb and Pugh, 2004; for review, see Weymouth and Vingrys, 2008). Figure 5, *A* and *B*, shows representative scotopic traces for retinas from *PV^Cre^*::*scFvOTX2^tg/o^* and *PV^Cre^* mice. The a-wave and b-wave implicit times and amplitudes were plotted against log intensity (Fig. 5*C–F*). Two-way ANOVA with repeated measures revealed no significant differences between the two genotypes (a-wave latency, *F*_(1,14)_ = 0.011; a-wave amplitude, *F*_(1,14)_ = 0.100; b-wave latency, *F*_(1,14)_ = 3.826; b-wave amplitude *F*_(1,14)_ = 0.77, all nonsignificant) or for genotype × stimulus intensity interaction (a-wave latency, *F*_(3,42)_ = 0.660; a-wave amplitude, *F*_(3,42)_ = 0.913; b-wave latency, *F*_(3,41)_ = 1.915; b-wave amplitude, *F*_(3,42)_ = 0.139; all nonsignificant). The absence of a difference in a-wave amplitude or implicit time in the *PV^Cre^*::*scFvOTX2^tg/o^* mice compared with *PV^Cre^* littermates indicates normal number and function of rod photoreceptors (Fig. 5; also see Fig. 7). The b-wave is mainly driven by bipolar activity, and the amplitude reflects the number and the implicit time bipolar function. Both were indistinguishable between *PV^Cre^*::*scFvOTX2^tg/o^* mice and *PV^Cre^* littermates, indicating the normal number and response of bipolar cells. The b-wave latency and amplitude under photopic conditions was similar in *PV^Cre^*::*scFvOTX2^tg/o^* mice and *PV^Cre^* littermates (Fig. 6). Interestingly, we observed a significant decrease in the amplitude in oscillatory potential (OP) 4 in *PV^Cre^*::*scFvOTX2^tg/o^* mice (Fig. 7*A*,*B*). There were no changes in earlier or later OPs. Additionally, for the flicker response, the *PV^Cre^*::*scFvOTX2^tg/o^* mice had an increase of ∼33% in amplitude at 10 Hz, and at 20 Hz the response significantly increased by twofold (Fig. 7*C*,*D*). Both of these results suggest inner retinal dysfunction at the level of amacrine cells and/or RGCs.

## Discussion

OTX2 homeoprotein is part of the HP family of transcription factors that transfer between cells and have neuroprotective effects in the brain (Sugiyama et al., 2008; Torero Ibad et al., 2011; Rekaik et al., 2015; for review, see Di Nardo et al., 2018). We were interested in assessing the importance of the direct non-cell autonomous activity of OTX2 in the retina since the Otx2 locus is silent in cells of the GCL, yet they contain OTX2 protein (Rath et al., 2007; Sugiyama et al., 2008). Since the amino acid sequences for OTX2 to exit and enter cells lie in the DNA binding homeodomain, mutations in these sequences might alter DNA binding and thus cell autonomous activity. Bernard et al. (2016) used a genetic approach to neutralize extracellular OTX2 by expressing a secreted anti-OTX2 single-chain antibody. Here, we used this approach to drive the expression of the OTX2-scFv in cells that do not themselves produce OTX2, thus eliminating a putative cell autonomous effect of the antibody. PV is reported to be in AII amacrines in rats, cats, bats, and rabbits (Lee et al., 2004). In the mouse, direct immunofluorescence showed low PV expression in amacrines and displaced amacrines, and strong PV expression in RGCs (Haverkamp and Wässle, 2000). A more sensitive transgenic approach using the PV promoter to drive expression of enhanced GFP (Haverkamp et al., 2009) confirmed this expression pattern and showed that PV was expressed in some amacrines in the INL with notably strong expression in RGCs (Haverkamp et al., 2009). Kim and Jeon (2006) and later Yi et al. (2012), using single-cell iontophoresis and retrograde tracing followed by PV localization, showed that at least eight different RGC types express PV. Thus, we anticipated widespread expression of the scFv throughout the GCL and inner INL. It is interesting to note that the ISH results showed scFv expression in fewer cells than expected, but it was sufficient to neutralize extracellular OTX2 and cause functional and vision changes.

Parvalbumin starts to be expressed in RGCs at approximately P11, after the birth of all retinal cell types (Young, 1985; Cepko et al., 1996). Thus, it is expected that interference with OTX2 non-cell autonomous activity starting at the earliest at P11 would have no effect in establishing the normal layers and structure of the retina, as we observe. It was reported that in *Otx2* hypomorph mice, in which both cell autonomous and non-cell autonomous phenotypes can be expected, there were fewer photoreceptors at P30, but those that were present have normal a-wave implicit time (Bernard et al., 2014). In *PV^Cre^*::*scFvOTX2^tg/o^* mice, the presence of normal numbers of OTX2-expressing photoreceptors and bipolar cells and their normal ERG a-wave and b-wave activity, respectively, confirms that OTX2 cell autonomous activity in these cells is not affected by expression of the secreted OTX2-scFv. It further suggests that OTX2 non-cell autonomous activity on these cells does not contribute measurably to their survival and function, at least at 1 month of age.

It is notable that we observed no changes in retinal structure or organization in the *PV^Cre^*::*scFvOTX2^tg/o^* mouse retina and that both ONL and INL function, at least in terms of ERG a- and b-wave activity, was comparable to *PV^Cre^* littermates, and yet the *PV^Cre^*::*scFvOTX2^tg/o^* mice had a deficit in visual acuity. How might this occur? This was not simply because of the expression of an scFv since *PV^Cre^*::*scFvPAX6^tg/o^* mice did not show any loss of visual acuity compared with *PV^Cre^* mice. PV is expressed in other regions of the nervous system and could interfere with OTX2 non-cell autonomous activity in these regions, thus contributing to the reduction of visual acuity. The optokinetic response does not depend on visual cortex since large lesions of posterior cortex do not diminish visual acuity, indicating that the brain circuits for this response are entirely subcortical (Douglas et al., 2005). The basic circuit for the optokinetic response is retinal projection to the nucleus of the optic tract that sends input to the reticular tegmental nucleus of the pons with output going to the vestibular nucleus driving the oculomotor nucleus (Wada et al., 2014). None of these structures are known to express OTX2, and only the vestibular nucleus has PV cells. Thus, the only structure in the basic circuit that has both PV and OTX2 expression is the retina.

We propose that the loss of non-cell autonomous signaling OTX2 induces inner retinal physiological dysfunction, as suggested by the altered OP and flickers response. OPs are an indication of inner retinal function and appear to be driven mainly by rod activity (Wachtmeister, 1998; Lei et al., 2006). OP ERG has been used to evaluate inner retina function in both human and experimental glaucoma (Gur et al., 1987; Rangaswamy et al., 2006). Early OPs have been attributed to GABAergic inhibitory activity in the ON pathway, while later OPs have been pharmacologically characterized as being generated by glycinergic inhibitory amacrine feedback synapses in the OFF pathway (Wachtmeister, 1998). Flickers reflect cone pathway activity (Tanimoto et al., 2015), and the cone photoreceptor function and cone bipolar function, as measured in the photopic a-wave and b-wave ERGs, is not compromised in the *PV^Cre^*::*scFvOTX2^tg/o^* retinas. Thus, the altered flicker responses that we observe represent a change in cone pathway activity at the level of the inner retina. Retinal ganglion cell dysfunction and degeneration in a model of glaucoma greatly alter the flicker response (Grozdanic et al., 2003), and the flicker ERG has been used to assess RGC function and hemodynamic changes in the mouse retina (Chou et al., 2019). The flicker response in macaques has been shown to be largely driven by spiking inner retinal neurons since it is increased at some frequencies by TTX and NMDA (Viswanathan et al., 2002). Here, we observed the same direction of change in *PV^Cre^*::*scFvOTX2^tg/o^* mice at 10 Hz, and significantly at 20 Hz. OPs and flickers are elicited by different stimuli. The OPs are part of the b-wave response elicited in our case by scotopic flash. The flickers are elicited by flicker stimuli in light-adapted conditions. Thus, two independent stimuli in different light adaptation conditions reveal inner retinal dysfunction when there is a decrease in OTX2 signaling in the retina.

We propose that non-cell autonomous OTX2 signaling in the inner retina is affected without loss of RGCs or amacrine cells in the GCL in the *PV^Cre^*::*scFvOTX2^tg/o^* mouse retina. What could be the nature of this signaling? Homeoproteins including OTX2 are capable of transferring between cells and, in addition to their transcriptional activity, can stimulate protein translation, alter chromatin remodeling, and repress transposable element expression (for review, see Blaudin de Thé et al., 2018; Di Nardo et al., 2018). OTX2 from the retina can reach inhibitory PV cells in V1 cortex and interference with its non-cell autonomous activity perturbs the normal opening and closing of the critical period for ocular dominance plasticity by altering the balance of excitation and inhibition (Sugiyama et al., 2008; Bernard et al., 2016). The change in flicker amplitude we recorded in the *PV^Cre^*::*scFvOTX2^tg/o^* mouse retina and OP may be related to a change in the excitation/inhibition of the inner retinal circuitry. It was reported that extracellular OTX2 neutralization in the retina can retard the opening of critical period plasticity in V1 cortex (Sugiyama et al., 2008). Since visual acuity measured by the optomotor test is independent of the visual cortex, this means that OTX2 signaling in the retina has separate physiological consequences both on subcortical and cortical visual circuits.

Non-cell autonomous signaling by the homeoproteins ENGRAILED 1/2 is important for RGC axon guidance in *Xenopus* and chick tectum, and this signaling involves stimulating ATP production and release from the RGC growth cone and subsequent activation of adenosine A1 receptor (Brunet et al., 2005; Wizenmann et al., 2009; Stettler et al., 2012). Retinal ganglion cells have particularly high energy demands, and energy homeostasis is critical for their function; and energy deficits are hypothesized to underlie RGC dysfunction and degeneration in diabetes, glaucoma, and optic neuropathies (Osborne and del Olmo-Aguado, 2013; Yu et al., 2013; Ito and Di Polo, 2017). Bipolar cell degeneration in *Otx2* hypomorph mice has been attributed to mitochondrial dysfunction that can be reversed by exogenous OTX2 (Bernard et al., 2014; Kim et al., 2015). While our finding could possibly be explained by a metabolic defect, it remains to be determined whether RGC mitochondrial energetics are altered in the *PV^Cre^*::*scFvOTX2^tg/o^* retina and whether this affects inner retina activity. Because displaced amacrine cells and RGCs in the GCL, major components of the inner retinal circuit, do not themselves express the *OTX2* gene but are capable of taking up exogenous OTX2 (Sugiyama et al., 2008; Torero Ibad et al., 2011), it can be proposed that interfering *in vivo* with OTX2 non-cell autonomous activity at the level of the mature retina leads to an alteration in inner retinal cell functions in early life and causes the observed deficit in visual acuity. Future studies will be required to establish whether these functional changes are longer lasting.

## Synthesis

Reviewing Editor: Siegrid Löwel, University of Goettingen Decisions are customarily a result of the Reviewing Editor and the peer reviewers coming together and discussing their recommendations until a consensus is reached. When revisions are invited, a fact-based synthesis statement explaining their decision and outlining what is needed to prepare a revision will be listed below. The following reviewer(s) agreed to reveal their identity: Karin Dedek.

The authors have addressed some of the concerns of the original reviews, but the main results of the behavioral tests and the ERG recordings remain weak, and the conclusions are based on indirect/inconclusive results.

The following concerns need to be addressed:

(1) Optomotor test: did the visual acuity of the experimental group change compared to the controls? So far, the authors have only tested responses at one spatial frequency (0.375cyc/deg), and only at P30. According to the authors, OTX2-scFv is turned on late at P11. What about other age groups, e.g. P60 and P100? What about contrast sensitivity? So far, the observed effect seems minor, and the authors need to do more work to convince readers it is something real.

(2) ERG recordings: Please also test different age groups, and test OPs to convince the readers that there are significant changes in inner retinal function. Please also explain the difference between 10 and 20 Hz flickers. So far both pieces of data are inconclusive and not convincing.

Right now, described differences are fairly small, and the evidence is indirect, so probably conclusions need to be downtoned.
